# Methodology and Initial Results From a Real-World Observational Cohort of Patients With Inflammatory Bowel Disease: TARGET-IBD

**DOI:** 10.1093/crocol/otab023

**Published:** 2021-05-13

**Authors:** Benjamin Click, Edward L Barnes, Benjamin L Cohen, Bruce E Sands, John S Hanson, Miguel Regueiro, David T Rubin, Marla C Dubinsky, Derek R Gazis, Laura Dalfonso, Janet S Hildebrand, Julie M Crawford, Millie D Long

**Affiliations:** 1 Department of Gastroenterology, Hepatology, and Nutrition, Cleveland Clinic, Cleveland, OH, USA; 2 Division of Gastroenterology and Hepatology, University of North Carolina at Chapel Hill, Chapel Hill, NC, USA; 3 Henry D. Janowitz Division of Gastroenterology, Icahn School of Medicine at Mount Sinai, New York, NY, USA; 4 Atrium Health Gastroenterology and Hepatology, Charlotte, NC, USA; 5 Inflammatory Bowel Disease Center, University of Chicago Medicine, Chicago, IL, USA; 6 Division of Pediatric Gastroenterology, The Susan & Leonard Feinstein IBD Clinical Center at Icahn School of Medicine at Mount Sinai, New York, NY, USA; 7 Target RWE, Inc., Durham, NC, USA

**Keywords:** Crohn disease, ulcerative colitis, real-world, comparative effectiveness, academic, community, registry

## Abstract

**Background:**

Data on care patterns for inflammatory bowel disease (IBD) from large-scale, diverse clinical cohorts in real-world practice are sparse. We developed a real-world cohort of patients receiving care at academic and community sites, for comparative study of therapies and natural history of IBD.

**Methods:**

We describe novel methodology of central abstraction of clinical data into a real-world IBD registry with patient reported outcomes (PROs). Baseline demographics, clinical characteristics, healthcare utilization, and disease metrics were assessed. Bivariate statistics were used to compare demographic and clinical data by Crohn disease (CD) or ulcerative colitis (UC) and site of care (academic, community).

**Results:**

In 1 year, 1343 IBD patients (60.1% CD, 38.9% UC) were recruited from 27 academic (49.5%) and community (50.5%) sites, exceeding expectations (110% enrolled). Most participants also consented to provide PROs (59.5%) or biosamples (85.7%). Overall, 48.7% of the cohort provided a baseline PRO, and 62.6% provided a biosample. Compared to UC, CD subjects had higher prior (34.1% CD vs 7.7% UC; *P* < 0.001) and current (72.1% vs 47.9%; *P* < 0.001) biologic utilization. CD participants from academic sites had more complicated disease than those from community sites (62.5% vs 46.8% stricturing/penetrating; 33.5% vs 27% perianal; 36.8% vs 14.5% prior biologic, respectively). Nearly all (90.4%) participants had endoscopic data of whom 37.7% were in remission. One-year retention was 98.4%.

**Conclusions:**

Centralized data abstraction and electronic PRO capture provided efficient recruitment into a large real-world observational cohort. This novel platform provides a resource for clinical outcomes and comparative effectiveness research in IBD.

## Introduction

An estimated 3.1 million individuals in the United States are affected by inflammatory bowel disease (IBD), including both Crohn disease (CD) and ulcerative colitis (UC).^1^ IBD significantly impacts quality of life, and can lead to substantial healthcare resource utilization. The care of IBD patients generates an estimated $14–31 billion in direct and indirect costs per year.^2^ Costs of care are unevenly distributed across the population as approximately 20% of patients with IBD account for 80% of all costs.^3^

While multiple biologic and small molecule therapies have been developed since the approval of first-in-class antitumor necrosis factor (TNF) biologic infliximab for the treatment of CD in 1998 and UC in 2005, our understanding of the appropriate sequencing of these therapies is limited. With a paucity of published data from randomized clinical trials (RCTs) on comparative effectiveness of biologic therapies, many decisions regarding first- and second-line biologic therapy use are primarily based on the experience of the treating clinician, opinion, experiential-based treatment algorithms, insurance or payer reimbursement, and shared-decision making with patients.

Although RCTs remain the highest level of evidence for the approval of new therapies, the majority of patients with IBD would not qualify for such clinical trials.^4^ Thus, the generalizability of the results from RCTs may only be applicable to a relatively narrow patient population. Real-world cohort studies offer an opportunity to evaluate health outcomes associated with novel therapies among patients with CD and UC, who have multiple comorbidities and varying degrees of compliance. Additionally, real-world observational studies allow for investigations of comparative effectiveness, safety, tolerability of newly developed therapies, and sequencing of treatment options among patients receiving care in usual clinical practice. Furthermore, observational studies allow for data to be collected on clinical effectiveness, the outcomes associated with agents in real-world practice, as compared to efficacy, which is a measure of outcomes under the narrow confines of RCTs.

Several novel concepts in the treatment of IBD have emerged in the past decade including more aggressive use of combination therapy,^5,6^ therapeutic drug monitoring,^7^ and treat-to-target management strategies.^8–11^ Additionally, an increased emphasis has been placed on the role that comorbid conditions, such as anxiety and depression, play in the disease course of patients with IBD.^12–15^ As new guidelines emerge to incorporate these shifts in the management of patients with both CD and UC,^14,16–21^ having a real-world cohort to evaluate changes in practice patterns is critical to assess the effectiveness of practice modifications and opportunities for further improvement. Thus, the aim of this longitudinal observational study is to establish a large cohort of patients treated for IBD at both academic and community sites across the United States to evaluate the course of disease in different populations and under different treatment conditions, and to develop evidence-based decisions for IBD-specific therapy in a large population of patients receiving care in real-world practice.

## Materials and Methods

TARGET-IBD is a longitudinal observational cohort study beginning in July 2017 of patients with IBD receiving medical care across academic (N = 21) institutions and community (N = 13) gastroenterology practice sites in the United States. The structure of TARGET-IBD is similar to that of other TARGET observational cohorts (TARGET-NASH, TARGET-HCC, TARGET-DERM, TARGET-PBC, TARGET-HBV) which aim to describe the real-world diagnosis, management, and disease course.^22–24^

### Inclusion Criteria

Adults (≥18 years) and children (2–17 years) with a diagnosis of CD, UC, or indeterminate colitis (inflammatory bowel disease undefined) as determined by a treating physician and who are receiving any prescription therapy for IBD are eligible for inclusion. Patients unable to provide written informed consent/assent, enrolled in any interventional study for IBD therapy, those with a history of prior total abdominal colectomy for UC, or those not meeting the inclusion criteria are excluded.

### Study Objectives

TARGET-IBD is a core resource for important collaborative translational studies utilizing biospecimens and clinical data from diverse patient populations. The primary and secondary aims of the study are listed in Table 1 and focus on evaluating the clinical effectiveness and safety of various treatment regimens for IBD. Data from the cohort may rapidly inform strategies to improve the management of patient populations that are underrepresented in clinical trials.

**Table 1.** Primary and secondary aims of TARGET-IBD

Primary aims	• Evaluate IBD treatment regimens being used in clinical practice
	• Examine outcomes of biosimilar use, including nonmedical switches from originator biologic, reverse switches back to the originator, and cross-switches between biosimilars
	• Examine populations underrepresented in phase III clinical trials
	• Evaluate optimal duration, timing, sequence, and combination of IBD therapy(ies) to achieve clinical response and clinical remission
	• Evaluate endoscopic outcomes
	• Estimate adverse event frequency and severity and describe management practices
	• Evaluate outcomes related to enrollment in, utilization of, and satisfaction with patient support programs
Secondary aims	• Describe response rates and safety in special populations
	• Evaluate drug–drug interactions
	• Evaluate health outcomes and durability of clinical response/clinical remission and time to relapse/treatment failure
	• Evaluate optimal dosing of therapy (eg, escalation of dosing of biologics)
	• Determine predictors of treatment response
	• Evaluate outcomes and durability of clinical response specifically among those with extraintestinal manifestations
	• Evaluate corticosteroid use
	• Evaluate malignancies
	• Evaluate opportunistic infections
	• Evaluate paradoxical reactions to therapies
	• Evaluate PROs measures
	• Evaluate surgeries and hospitalizations

### Study Procedures and Ethical Considerations

Up to 15,000 participants with IBD, including both adult and pediatric populations, will be enrolled in TARGET-IBD. Clinical patient management follows each site’s local standard of care, and no specific treatments, clinical assessments, or laboratory tests potentially influencing care are dictated by enrollment. Central and/or local institutional review board (IRB) approvals are obtained prior to enrollment. The entire study is conducted in accordance with good clinical practice requirements and compliance with the ethical principles described in the current revision of the Declaration of Helsinki. A transparent informed consent form is given to potential participants by their clinician; this form explains that their information will be used for the study, but also may be shared with others, including persons, agencies, or companies that enter into a contract with Target RWE to have access to such information. There is a biorepository collection consent that is an optional component of the study, and its consent spells out that samples may be used for research, and that biospecimen information may also be shared with companies contracting with the study sponsor. Throughout the study, all information provided to industry partners is given in an aggregated or deidentified manner to further protect study participants and ensure data sharing is done ethically and in compliance with participant consent.

Consented patients provide up to 3 years of retrospective, redacted medical information and are then followed prospectively for up to 5 years (Table 2). There are no study-mandated interventions or assessments and clinical follow-up is determined by the treating physician. Redacted medical records from participating sites are uploaded every 6 months into a secured data repository. Central abstractors then collect information on patient demographics, comorbid conditions, disease characteristics, medication use, healthcare utilization, imaging tests, endoscopic procedures, surgical reports, pathology reports, and laboratory tests.

**Table 2.** Table of procedures for TARGET-IBD activity

Activity	Screening/enrollment visit*					Follow-up															
	Year 1					Year 2				Year 3				Year 4				Year 5			
	Month 0	3	6	9	12	15	18	21	24	27	30	33	36	39	42	45	48	51	54	57	60
Informed consent	X																				
Inclusion/exclusion	X																				
Blood sample collection^†^	X				X				X				X				X				X
PRO surveys^‡^	X	X	X	X	X	X	X	X	X	X	X	X	X	X	X	X	X	X	X	X	X
Medical records submission^¶^	X^§^	X	X	X	X	X	X	X	X	X	X	X	X	X	X	X	X	X	X	X	X

*Study procedures will be completed at a regularly scheduled clinic visit.

^†^Participants will be asked to provide an optional blood sample.

^‡^Participants will be asked to complete optional PRO measures every 3 months.

^§^Three years of redacted historical records may be submitted following the Screening/Enrollment visit.

^¶^During the follow-up period, redacted medical records will be submitted every 3 months for up to 5 years. The first submission during the follow-up period will be 3 months following the Screening/Enrollment visit. Additional interim medical records submissions may be requested.

All data are stored centrally by an electronic data capture system. Clinical monitors review select data to ensure entered data correspond with data in the source document (ie, redacted medical records). Coding is performed as appropriate using MedDRA and WHODrug dictionaries.

Patients who enroll in an interventional clinical trial at any point during follow-up are flagged and data collection is temporarily put on hold for future reactivation. Patients who withdraw have future data collection cease, but existing or retrospective data along with biospecimens remain in the database and/or repository, respectively. Patients who transfer care to another TARGET-IBD center may continue participation.

### Exposure Measures

Data are collected on demographics, comorbidities, disease course, disease phenotype, prior IBD-related surgeries and hospitalizations, as well as medication utilization.

### Outcome Measures

Endoscopic and histologic data are collected as part of routine care. When available, endoscopic scores are abstracted from clinical documentation, including the Mayo score for UC and the simple endoscopic score (SES-CD) for CD. If scoring is not available in documentation, descriptors used in the endoscopic reporting are captured, including remission/normal, mild, moderate, or severe inflammation. These classifications have allowed all endoscopic reports to be classified as active (any inflammation > normal) or inactive (normal/remission). Histologic inflammatory activity data are also collected and categorized by inflammation presence, histologic activity (mild, moderate, or severe based on highest level of activity reported), evidence of chronicity, and presence of dysplasia.

### Patient Reported Outcomes

Patients enrolled in TARGET-IBD have the option of completing patient reported outcome (PRO) measures as well as providing blood samples for the biospecimen repository. Participation in the collection of these additional measures does not affect participation in the main cohort.

Patients who consent are asked to complete the measures at 3-month intervals either on paper or via a web-based system. The PRO measures employed include quality of life, disease activity, and medication adherence questionnaires (Table 3). Validated disease activity measures include the Manitoba IBD index^25^ and the pediatric UC activity index (PUCAI).^26^ Bowel movement frequency, urgency, rectal bleeding, and other components in the PRO-2 for CD and UC are also collected.^27,28^ Data are also abstracted from clinical documentation, where providers mention bowel movement frequency.

**Table 3.** PROs employed in TARGET-IBD

PRO	Domain	Condition	Ages
EuroQol-5D-5L^29^	Quality of life	CD, UC, IBDU	Adult
PRO-2^27^	Disease activity	CD	Adult, pediatric
PRO-2^28^	Disease activity	UC	Adult
PUCAI^26^	Disease activity	UC	Pediatric
MIBDI^25^	Disease activity	CD, UC, IBDU	Adult
Adherence measure^30^	Medication adherence	CD, UC, IBDU	Adult

IBDU, inflammatory bowel disease undefined; MIBDI, Manitoba inflammatory bowel disease index.

### Biorepository Samples

Consent for biospecimens (whole blood and serum) is obtained at enrollment and specimens are collected prospectively at 12-month intervals (Table 2). These samples are shipped to a central repository for storage to use in future analyses. Obtaining samples for biomarkers or DNA at a given site are contingent on that site’s IRB regulations. Collected samples will be stored indefinitely and may be used for teaching as well as research toward the development of new medical products or diagnostics tests relevant to IBD.

### Statistical Analysis

In the initial report of baseline data, bivariate analyses were used to compare characteristics by disease subgroup (CD or UC) and by site of care (community vs academic practice). Rates of completion of PRO data were calculated, as well as initial disease activity classification by PROs and by endoscopic outcome data. Rates of completion of biosample collection were also calculated. All analyses were completed using SAS (version 9.4) statistical software (SAS Institute, Cary, NC).

### Role of Sponsor

TARGET-IBD receives funding from multiple industry partners. TARGET PharmaSolutions conducts the work under the guidance of a Steering Committee consisting of gastroenterology thought leaders. Research proposals can be suggested by participating site principle investigators, Steering Committee members, Publication Committee members, and industry partners; they are in turn reviewed for merit and feasibility by the Publications Committee (consisting of Steering Committee members and several other gastroenterologists). TARGET PharmaSolutions executed a Cooperative Research and Development Agreement (CRADA #299-17) with the Food and Drug Administration (FDA) and has periodic meetings at FDA to discuss the cohort’s data.

## Results

### Year-One Enrollment

**Figure 1.**
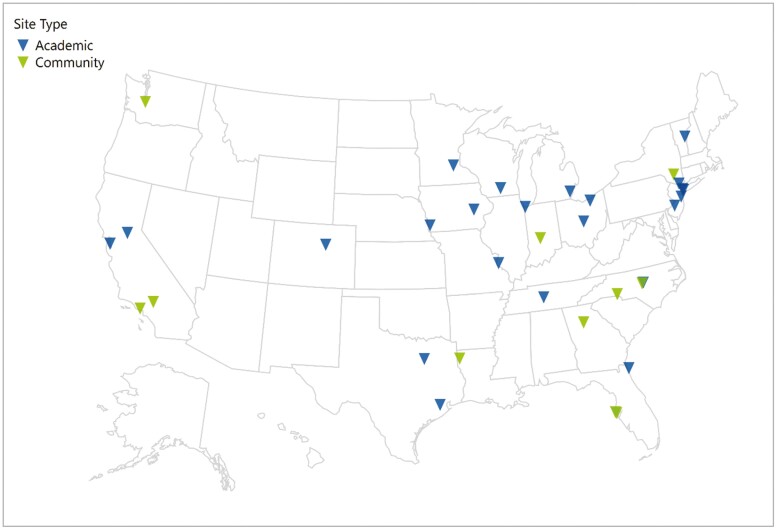
Map of currently enrolling sites for TARGET-IBD.

**Figure 2.**
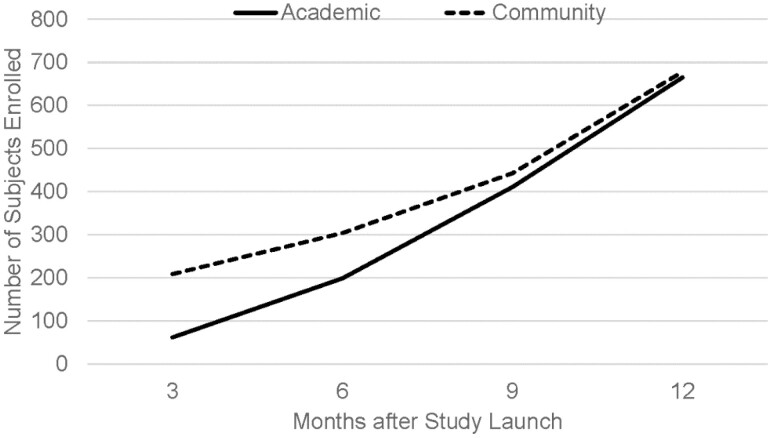
Rate of TARGET-IBD subject enrollment over the first year by site of care.

Novel methodology of centralized abstraction and electronic PRO reporting resulted in recruitment of 1343 IBD patients in 1 year from 27 sites; data abstraction was completed for 997 (74.2%) by the end of the year (Fig. 1). The majority (60.1%) had CD, were female (54.7%), and Caucasian (86.6%). Median age at study entry was 45 years with predominance of adult participants (99.3%). Site of care was relatively equally represented, with 49.5% (n = 494) of the population recruited from academic sites and 50.5% (n = 503) from community sites (Fig. 2). Special populations of interest included elderly IBD (age >65 years) (n = 151, 15.2%), perianal disease (n = 226, 22.7%), and active inflammatory extraintestinal manifestations (n = 77, 7.7%). Over the first year, only 16 (1.6%) participants discontinued with the predominant reasons of interventional trial enrollment (n = 6) or subject withdrawal (n = 6).

In the 3-year retrospective period, 21,491 unique healthcare encounters were identified, including 2146 endoscopic procedures, 994 radiography studies, and 6176 clinic visits. Nearly all patients (90.4%) had at least 1 endoscopic assessment; including only a participant’s most recent endoscopy, 37.7% were interpreted as normal. Overall, nearly half (48.7%) of the year-one cohort consented to and completed at least 1 baseline PRO assessment. A total of 62.6% consented to and provided biosamples.

### Differences by Disease Type

Compared to UC subjects, the median disease duration at the time of enrollment was significantly longer in the CD participants (11 years CD vs 8 years UC; *P* < 0.001) (Table 4). There were more active smokers in CD than UC (11.6% vs 3.8%, respectively, *P* < 0.001). Significantly more CD subjects were enrolled at academic sites than community (*P* = 0.006). CD participants had significantly higher rates of previous biologic exposure (34.1%) than UC (7.7%) (*P* < 0.001). Furthermore, during the retrospective and prospective study period, those with CD had a greater rate of advanced therapy use, with 72.1% on biologics or small molecules compared to 47.9% UC (*P* < 0.001). For both CD and UC, the most utilized class of biologics was anti-TNFs (88.0% CD, 83.9% UC).

**Table 4.** Baseline demographics, disease characteristics, treatment details, and TARGET-IBD activity for the year-one study cohort

	All (N = 997)	UC (n = 388)	CD (n = 599)	IBDU (n = 10)	*P* (CD vs UC)
Age at study entry (years)					
Median (n)	45 (992)	47 (385)	44 (597)	37 (10)	0.0137
Q1–Q3 (IQR)	32–59 (27)	34–61 (27)	30–59 (29)	31–47 (16)	
Female sex, n (%)	545 (54.7)	203 (52.3)	339 (56.6)	3 (30)	0.1876
Race, n (%)					0.0946
White	832 (86.6)	325 (87.4)	499 (86.2)	8 (80.0)	
Black	86 (8.9)	26 (7.0)	59 (10.2)	1 (10.0)	
Other	43 (4.5)	21 (5.6)	21 (3.6)	1 (10.0)	
Not available	36	16	20	0	
BMI (kg/m^2^)					
Median (n)	26 (954)	27 (376)	26 (569)	33 (9)	0.0051
Q1–Q3 (IQR)	23–31 (8)	24–32 (8)	22–30 (8)	25–42 (17)	
Current smoker, n (%)	82 (8.6)	14 (3.8)	67 (11.6)	1 (10.0)	<0.0001
Not available	43	20	23	0	
Site type, n (%)					
Academic	494 (49.5)	154 (39.7)	333 (55.6)	7 (70.0)	<0.0001
Community	503 (50.5)	234 (60.3)	266 (44.4)	3 (30.0)	
Disease duration (years)					
Median (n)	10 (877)	8 (342)	11 (525)	8 (10)	<0.0001
Q1–Q3 (IQR)	4–18 (14)	3–14 (11)	5–22 (17)	3–16 (13)	
CD location, n (%)	599	0	599	0	—
Colon	114 (19.8)	NA	114 (19.8)	NA	
Ileocolonic	336 (58.4)	NA	336 (58.4)	NA	
Ileum	125 (21.7)	NA	125 (21.7)	NA	
Not available	24	NA	24	NA	
Perianal disease, n (%)	154 (30.6)	0 (NA)	154 (30.6)	0 (NA)	—
Not available	95	NA	95	NA	
CD behavior, n (%)	544	0	544	0	—
Inflammatory	241 (44.3)	NA	241 (44.3)	NA	
Stricturing	149 (27.4)	NA	149 (27.4)	NA	
Penetrating	154 (28.3)	NA	154 (28.3)	NA	
Not available	55	NA	55	NA	
UC extent, n (%)	361	361	0	0	—
Extensive	176 (48.8)	176 (48.8)	NA	NA	
Left-sided	139 (38.5)	139 (38.5)	NA	NA	
Proctitis	46 (12.7)	46 (12.7)	NA	NA	
Not available	27	27	NA	NA	
Prior biologics, n (%)					
Anti-TNF	235 (23.6)	29 (7.5)	204 (34.1)	2 (20)	<0.0001
Anti-integrin*	16 (1.6)	4 (1.0)	12 (2.0)	0 (0.0)	0.2376
Ustekinumab	4 (0.4)	1 (0.3)	3 (0.5)	0 (0.0)	0.5573
Medications during study, n (%)^†^					
5-ASA	551 (55.3)	339 (87.4)	206 (34.4)	6 (60.0)	<0.0001
IM^‡^	406 (40.7)	128 (33.0)	275 (45.9)	3 (30.0)	<0.0001
Steroids^§^	568 (57.0)	243 (62.6)	315 (52.6)	10 (100.0)	0.0019
Anti-TNF	539 (54.1)	156 (40.2)	380 (63.4)	3 (30.0)	<0.0001
Vedolizumab	155 (15.5)	69 (17.8)	85 (14.2)	1 (10.0)	0.1288
Ustekinumab	61 (6.1)	2 (0.5)	59 (9.8)	0 (0.0)	<0.0001
Tofacitinib	1 (0.1)	1 (0.3)	0 (0.0)	0 (0.0)	0.2141
Healthcare encounter count during study, n^†^					
Endoscopy	2146	758	1361	27	
Imaging	994	144	841	9	
IBD surgery during study, n (%)^†^	260 (26.1)	11 (2.8)	249 (41.6)	0 (0.0)	<0.0001
At least 1 PRO completed, n (%)	562 (56.4)	208 (53.6)	351 (58.6)	3 (30.0)	0.1226
Participation ongoing, n (%)	981 (98.4)	376 (96.9)	595 (99.3)	10 (100.0)	0.0032

*Anti-integrin data include natalizumab and vedolizumab.

^†^Includes both the 3-year retrospective period as well as prospective data collection.

^‡^Includes azathioprine, mercaptopurine, or methotrexate.

^§^Includes prednisone, hydrocortisone, methylprednisolone, and budesonide.

5-ASA, 5-aminosalicylate; BMI, body mass index; IBDU, inflammatory bowel disease undefined; IM, immunomodulator; IQR, interquartile range; TNFa, tumor necrosis factor alpha.

Similar proportions of participants underwent endoscopy in UC (90.7%) and CD (90.2%) and there were similar rates of endoscopic remission between UC (36.2%) and CD (38.9%). Radiographic studies were utilized more commonly in CD (59.7%) than UC (21.7%). There was increased use of MRI compared to other forms of imaging in CD (29.6%) compared to UC (16.9%) (*P* = 0.019).

### Variation by Site of Care

The median age of enrolled patients was younger in academic sites (median 40 years) compared to community sites (median 49 years), yet median disease duration was significantly longer (12.0 vs 8.0 years, respectively, *P* < 0.001) (Table 5). Academic sites had slightly more racially diverse participants. Academic-enrolled CD participants more commonly had stricturing (31.9%) or penetrating (30.6%) disease, compared to predominantly inflammatory disease (53.2%) at community sites. There was slightly more perianal involvement at academic (33.5%) vs community (27.0%) sites. UC extent was similar between enrollment locations. Prior biologic use was significantly more common in academic site participants (33.4%) than community subjects (14.1%, *P* < 0.001).

**Table 5.** Baseline demographics, disease characteristics, treatment details, and TARGET-IBD activity for the year-one study cohort by site of enrollment

	Community (n = 503)	Academic (n = 494)	*P*
Age at study entry (years)			
Median (n)	49 (503)	40 (489)	<0.0001
Q1–Q3 (IQR)	34–63 (29)	30–55 (25)	
Female sex, n (%)	273 (54.3)	272 (55.1)	0.8032
Race, n (%)			
White	435 (90.8)	397 (82.4)	0.0006
Black	28 (5.8)	58 (12.0)	
Other	16 (3.3)	27 (5.6)	
Not available	24	12	
BMI (kg/m^2^)			
Median (n)	27 (498)	26 (456)	0.1748
Q1–Q3 (IQR)	23–31 (8)	22–31 (9)	
Current smoker, n (%)	49 (10.1)	33 (7.0)	0.0840
Not available	20	23	
Disease duration (years)			
Median (n)	8 (404)	12 (473)	<0.0001
Q1–Q3 (IQR)	3–16 (13)	6–21 (15)	
CD location, n (%)			
Colon	57 (22.7)	57 (17.6)	0.2953
Ileocolonic	143 (57.0)	193 (59.6)	
Ileum	51 (20.3)	74 (22.8)	
Not available	15	9	
Upper GI tract disease, n (%)	33 (15.4)	62 (22.9)	0.0401
Not available	52	62	
Perianal disease, n (%)	1 (27.0)	93 (33.5)	0.1176
Not available	40	55	
CD behavior, n (%)			
Inflammatory	126 (53.2)	115 (37.5)	0.0009
Stricturing	51 (21.5)	98 (31.9)	
Penetrating	60 (25.3)	94 (30.6)	
Not available	29	26	
UC extent, n (%)			
Extensive	103 (47.9)	73 (50.0)	0.9255
Left-sided	84 (39.1)	55 (37.7)	
Proctitis	28 (13.0)	18 (12.3)	
Not available	19	8	
Prior biologics, n (%)			
Anti-TNF	71 (14.1)	164 (33.2)	<0.0001
Anti-integrin*	2 (0.4)	14 (2.8)	0.0022
Ustekinumab	0 (0.0)	4 (0.8)	0.0433
Medications during study, n (%)^†^			
5-ASA	305 (60.6)	246 (49.8)	0.0006
IM^‡^	149 (29.6)	257 (52.0)	<0.0001
Steroids^§^	284 (56.5)	284 (57.5)	0.7430
Anti-TNF	254 (50.5)	285 (57.7)	0.0227
Vedolizumab	73 (14.5)	82 (16.6)	0.3636
Ustekinumab	12 (2.4)	49 (9.9)	<0.0001
Tofacitinib	0 (0.0)	1 (0.2)	0.3129
Healthcare encounter count during study, n^†^			
Endoscopy	942	1204	
Imaging	389	605	
IBD surgery during study, n (%)	111 (22.1)	149 (30.2)	0.0036
At least 1 PRO completed, n (%)	248 (49.3)	314 (63.6)	<0.0001
Participation ongoing (%)	491 (97.6)	490 (99.2)	0.0478

*Natalizumab or vedolizumab.

^†^Includes both the 3-year retrospective period as well as prospective data collection.

^‡^Includes azathioprine, mercaptopurine, or methotrexate.

^§^Includes prednisone, hydrocortisone, methylprednisolone, and budesonide.

Biologic use during retrospective and prospective study periods was similar in academic and community sites for CD (73.9% academic vs 69.9% community, *P* = 0.28), whereas significantly higher proportions of UC patients seen in academic sites used biologics (57.1% vs 41.9%, *P* = 0.003). There was a similar rate of mesalamine use for CD patients at community and academic sites (34.6% vs 34.2%, *P* = 0.93).

The utilization of endoscopy was similar in both UC (90.6% community vs 90.9% academic, *P* = 0.92) and CD (91.0% community vs 89.5% academic, *P* = 0.54) by site of care. The proportion of endoscopic remission was greater in academic participants than community for both UC (42.4% vs 32.1%, respectively, *P* = 0.049) and CD patients (41.8% vs 35.4%, respectively, *P* = 0.13). Radiographically, there was increased utilization of MRI compared to other forms of imaging in academic participants with CD (34.1% academic vs 22.7% community; *P* = 0.021). Consent to complete PRO measures was given by the majority of participants (81.8% of those at academic sites and 82.1% of those at community sites). Baseline PRO measures were completed by 71.0% of those who consented from academic sites and 48.2% from community sites; at month 3, these values were 34.4% and 33.6%; at month 6, these values were 36.0% and 35.2%. Finally, consent to give biorepository samples was given by 73.0% of participants; of those, 85.7% provided at least 1 sample.

## Discussion

TARGET-IBD is a longitudinal observational study conducted across both academic and community sites which creates a real-world view of the natural history and clinical management of patients with IBD. Utilizing novel registry methods including centralized data abstraction and electronic PROs has allowed TARGET-IBD to surpass enrollment expectations and rapidly build a sizeable, representative IBD cohort. The data ascertained from participating subjects provide valuable information on the clinical effectiveness of IBD-related interventions. Thus, TARGET-IBD represents a powerful prospective cohort to address critical questions in IBD.

We observed several unique features of the year-one TARGET-IBD cohort. First, enrollment was rapid and exceeded expectations. Enrollment in TARGET-IBD during the first year was projected to be 1067 participants. Actual recruitment swiftly exceeded estimates (as year one ended with 1178 enrolled). Potential explanations for the rapid recruitment include broad eligibility criteria, minimal patient study requirements, centralized data abstraction, and electronic PROs. The study design allows for streamlined integration of coordinated screening and efficient recruitment in routine clinical practice, while ensuring a representative sample of real-world academic and community IBD patients. Compared to other observational IBD registries that may require significantly structured data entry or case report forms, TARGET-IBD’s use of centralized data abstraction from standard of care documentation reduces the clinician data entry requirements and facilitates subject enrollment. Furthermore, centralized abstraction via trained abstractors with quality assurance likely improves data consistency compared to data sources that rely on individual site interpretation and input. The utilization and completion of electronic PROs similarly expedites the data collection process, streamlines efficiency, and minimizes research burden. Further evidence of this enrollment and data efficiency is the continued steady rate of enrollment and high retention rate (4089 participants have enrolled in TARGET-IBD in the first two and a half years; 94.7% of the consented subjects are continuing to participate in the study, with median study duration of 16 months.

Second, there was nearly equal enrollment between community and academic centers. This participant distribution allows for a more representative sample of IBD care. Compared to other prospective registries, which often overly or uniformly recruit from academic centers,^31–34^ TARGET-IBD is maximizing generalizability by engaging a breadth of enrollment sites. The reliance on and overrepresentation of academic center patients in other registries is likely a product of investigator and patient interest, research experience and infrastructure, and interaction with the logistics of clinical care.

However, there were differences by site of enrollment. We observed variability in certain demographics, disease history and characteristics, biologic utilization, endoscopic remission rates, and imaging study selection by site of care. In the current cohort, patients cared for in academic centers had more complex CD and comorbidities, which is likely reflective of referral bias. Likely consequently, we observed higher rates of biologic utilization in academic participants with CD. Prior studies have demonstrated practice variability by setting^35^ as well as within-setting variation.^36^ To date, studies have not demonstrated any significant association of healthcare outcomes in IBD by site of care^37,38^; however, in the current data, we did observe higher rates of endoscopic remission in academic participants compared to community suggesting a potential influence of care variation. These prior works examining differences by academic vs community location relied on large administrative datasets. Such datasets rely on billing codes to identify cohorts and outcomes. They provide significant power to assimilate study populations and detect differences, but there are inherent flaws including misclassification bias, extensive confounding, and lack of clinical outcome data.^39,40^ Granular detail with individual-level data is needed to assess these limitations, though they may be addressed by the rapidly growing and increasingly representative population recruited in TARGET-IBD and may provide key insights into practice patterns and their outcomes.

TARGET-IBD recruited a slight predominance of CD compared to UC patients. This is similar to other US cohorts, both community and academic, such as the community-based OSCCAR (Ocean State Crohn’s and Colitis Area Registry)^41^ inception cohort,^42,43^ the SHARE (Sinai-Helmsley Alliance for Research Excellence) cohort, and IBD Partners,^44^ which all have a greater proportion of CD participants. The predominance seen across these cohorts may be related to disease-specific patient or provider research enrollment biases, referral bias of CD patients to providers conducting research, involvement with an advocacy organization such as the Crohn’s & Colitis Foundation, and other factors associated with survey response or participation bias.

In the current study, nearly two-thirds of UC and CD patients had active endoscopic disease despite therapy. As therapeutic goals have shifted toward mucosal improvement due to its association with disease outcomes,^45^ it is important to understand the achievability and influencing factors of this outcome. The prevalence of endoscopic healing in the current data is similar to that reported in other studies including both clinical trials and real-world outcomes,^46^ demonstrating that a minority of patients achieve this outcome, even with biologics. A prospective, longitudinal, real-world cohort will help illuminate predictive factors for inducing and maintaining such outcomes.

In addition, this study recruited both pediatric and adult participants. Though pediatric representation was low and prevented meaningful analysis, through targeted site selection and recruitment, there is now improved pediatric enrollment in TARGET-IBD. The main barriers initially to recruitment of younger patients was that very few of the original sites in the study recruited exclusively pediatric patients, and many sites did not care for pediatric patients. Other potential barriers included lower prevalence of pediatric IBD, more streamlined adult IBD research infrastructure in both community and academic practices, and hesitancy to enroll in clinical research by pediatric patients, parents, or guardians. Future studies will assess this growing pediatric IBD population.

There are other existing mechanisms, registries, and consortiums collecting real-world data. Other registry data sources include postmarketing surveillance cohorts [Janssen-sponsored TREAT Registry (NCT00553176)^47^ for infliximab, AbbVie-sponsored adalimumab in UC registry (NCT01848561), UCB-sponsored certolizumab pegol registry (NCT00844285), to name a few]. These cohorts typically rely on academic centers for enrollment, can be slow to recruit, and can span a significant number of years. The external generalizability of these results may be questionable, given the preponderance of academic sites involved in data collection. Similarly, there are multiple consortium groups combining retrospective data on a variety of IBD patient populations^32,33^; however, these studies often or variably lack the prospective data elements of PROs, almost universally depend on academic centers, submitting to selection or referral bias and confounding, and are prone to many retrospective biases.

The use of real-world data is gaining attention by regulatory authorities to support changes in drug labeling and effectiveness, as such data may be utilized as a “fit for use” source to generate real-world evidence to support regulatory submissions. These data will also be valuable for monitoring the frequency of drug-related adverse events and evaluating the impact of new interventions on health outcomes. TARGET-IBD may overcome some of the challenges of other data sources through its unique methodology. The rapid recruitment and streamlined data collection allow for evaluation of emerging practice patterns, recently approved therapies, or developing trends with only minimal delay, thus facilitating timely analyses and results dissemination.

The strengths of this study include the rapid recruitment via integration into the practice model utilizing centralized abstraction with imbedded quality assurance, the diverse and representative population, the specific level and granularity of data (including histologic activity), prospective PROs, and optional biorepository for future translational studies.

There are also limitations of this study. TARGET-IBD captures all records from the treating gastroenterologist and electronic medical record; however, it is possible that individual patients also receive care or laboratory testing outside of these which is not being captured. TARGET-IBD is collecting consent to link to administrative data sources to account for this potential for other care sites in the future. Participation in PRO and biorepository components is voluntary and thus not available for all participants. It must be recognized that reliance on existing clinical data may invite a larger degree of missing information or variability potentially introducing biases. Certain data elements are only available if routinely employed in clinical practice; thus, endoscopic scoring data are not universally available. However, TARGET-IBD employs data review and stratification based on available evidence. While this classification would not be used in RCTs, it is likely appropriate for studies of clinical effectiveness. There is limited racial or ethnic diversity thus far in the study cohort. This may be addressed in the future by targeted enrollment of underrepresented populations.

## Conclusions

TARGET-IBD is a large cohort of patients receiving standard care for IBD across academic and community sites in the United States. Clinical information on patients enrolled in the study is ascertained using standardized data collection practices, and the study data are monitored for quality and completeness. Moreover, the data are captured according to a comprehensive observational protocol to increase the efficiency of performing clinical research while ensuring collection of detailed critical safety and effectiveness data on prescribed IBD therapies. TARGET-IBD engages community and academic practice providers as partners in research to ensure rapid translation of research findings into improvement in healthcare quality and outcomes. Furthermore, the availability of an established, cohesive research network allows nimble responses to investigations of new treatment paradigms with existing agents, as well as future generations of IBD therapies.

## Data Availability

Data are not publicly available.

## References

[1] Dahlhamer JM , Zammitti EP, Ward BW, Wheaton AG, Croft JB. Prevalence of inflammatory bowel disease among adults aged ≥18 years — United States, 2015. MMWR Morb Mortal Wkly Rep. 2016;65:1166–1169. doi:10.15585/mmwr.mm6542a3 27787492

[2] Mehta F . Report: economic implications of inflammatory bowel disease and its management. Am J Manag Care. 2016;22:s51–s60. 27269903

[3] Kinnucan J , Binion D, Cross R, et al. Inflammatory bowel disease care referral pathway. Gastroenterology. 2019;157:242–254.e6. 30980795 10.1053/j.gastro.2019.03.064

[4] Ha C , Ullman TA, Siegel CA, et al. Patients enrolled in randomized controlled trials do not represent the inflammatory bowel disease patient population. Clin Gastroenterol Hepatol. 2012;10:1002–1007; quiz e78. 22343692 10.1016/j.cgh.2012.02.004

[5] Colombel JF , Sandborn WJ, Reinisch W, et al. Infliximab, azathioprine, or combination therapy for Crohn’s disease. N Engl J Med. 2010;362:1383–1395. 20393175 10.1056/NEJMoa0904492

[6] Panaccione R , Ghosh S, Middleton S, et al. Combination therapy with infliximab and azathioprine is superior to monotherapy with either agent in ulcerative colitis. Gastroenterology. 2014;146:392–400.e3. 24512909 10.1053/j.gastro.2013.10.052

[7] Papamichael K , Cheifetz AS, Melmed GY, et al. Appropriate therapeutic drug monitoring of biologic agents for patients with inflammatory bowel diseases. Clin Gastroenterol Hepatol. 2019;17:1655–1668.e3. 30928454 10.1016/j.cgh.2019.03.037 PMC6661210

[8] Peyrin-Biroulet L , Sandborn W, Sands BE, et al. Selecting Therapeutic Targets in Inflammatory Bowel Disease (STRIDE): determining therapeutic goals for treat-to-target. Am J Gastroenterol. 2015;110:1324–1338. 26303131 10.1038/ajg.2015.233

[9] Colombel JF , Panaccione R, Bossuyt P, et al. Effect of tight control management on Crohn’s disease (CALM): a multicentre, randomised, controlled phase 3 trial. Lancet. 2017;390:2779–2789. 29096949 10.1016/S0140-6736(17)32641-7

[10] Ungaro R , Colombel JF, Lissoos T, et al. A treat-to-target update in ulcerative colitis: a systematic review. Am J Gastroenterol. 2019;114:874–883. 30908297 10.14309/ajg.0000000000000183 PMC6553548

[11] Colombel JF , D’haens G, Lee WJ, Petersson J, Panaccione R. Outcomes and strategies to support a treat-to-target approach in inflammatory bowel disease: a systematic review. J Crohns Colitis. 2020;14(2):254–266. 31403666 10.1093/ecco-jcc/jjz131 PMC7008150

[12] Barnes EL , Kochar B, Long MD, et al. Modifiable risk factors for hospital readmission among patients with inflammatory bowel disease in a nationwide database. Inflamm Bowel Dis. 2017;23:875–881. 28426473 10.1097/MIB.0000000000001121 PMC5512697

[13] Kochar B , Barnes EL, Long MD, et al. Depression is associated with more aggressive inflammatory bowel disease. Am J Gastroenterol. 2018;113:80–85. 29134965 10.1038/ajg.2017.423 PMC5962285

[14] Farraye FA , Melmed GY, Lichtenstein GR, et al. ACG clinical guideline: preventive care in inflammatory bowel disease. Am J Gastroenterol. 2017;112:241–258. 28071656 10.1038/ajg.2016.537

[15] Mikocka-Walus A , Pittet V, Rossel JB, et al. Symptoms of depression and anxiety are independently associated with clinical recurrence of inflammatory bowel disease. Clin Gastroenterol Hepatol. 2016;14:829–835.e1. 26820402 10.1016/j.cgh.2015.12.045

[16] Rubin DT , Ananthakrishnan AN, Siegel CA, et al. ACG clinical guideline: ulcerative colitis in adults. Am J Gastroenterol. 2019;114:384–413. 30840605 10.14309/ajg.0000000000000152

[17] Lichtenstein GR , Loftus EV, Isaacs KL, et al. ACG clinical guideline: management of Crohn’s disease in adults. Am J Gastroenterol. 2018;113:481–517. 29610508 10.1038/ajg.2018.27

[18] Feuerstein JD , Isaacs KL, Schneider Y, et al. AGA clinical practice guidelines on the management of moderate to severe ulcerative colitis. Gastroenterology. 2020;158:1450–1461. 31945371 10.1053/j.gastro.2020.01.006 PMC7175923

[19] Ko CW , Singh S, Feuerstein JD, et al. AGA clinical practice guidelines on the management of mild-to-moderate ulcerative colitis. Gastroenterology. 2019;156:748–764. 30576644 10.1053/j.gastro.2018.12.009 PMC6858922

[20] Terdiman JP , Gruss CB, Heidelbaugh JJ, et al. American Gastroenterological Association Institute guideline on the use of thiopurines, methotrexate, and anti-TNF-α biologic drugs for the induction and maintenance of remission in inflammatory Crohn’s disease. Gastroenterology. 2013;145:1459–1463. 24267474 10.1053/j.gastro.2013.10.047

[21] Feuerstein JD , Nguyen GC, Kupfer SS, et al. American Gastroenterological Association Institute guideline on therapeutic drug monitoring in inflammatory bowel disease. Gastroenterology. 2017;153:827–834. 28780013 10.1053/j.gastro.2017.07.032

[22] Mishra P , Florian J, Peter J, et al. Public-private partnership: targeting real-world data for hepatitis C direct-acting antivirals. Gastroenterology. 2017;153:626–631. 28757271 10.1053/j.gastro.2017.07.025

[23] Barritt ASt , Gitlin N, Klein S, et al. Design and rationale for a real-world observational cohort of patients with nonalcoholic fatty liver disease: the TARGET-NASH study. Contemp Clin Trials. 2017;61:33–38. 28735109 10.1016/j.cct.2017.07.015

[24] Levy C , Bowlus CL, Carey E, et al. A real-world observational cohort of patients with primary biliary cholangitis: TARGET-primary biliary cholangitis study design and rationale. Hepatol Commun. 2018;2:484–491. 29761165 10.1002/hep4.1173 PMC5944592

[25] Clara I , Lix LM, Walker JR, et al. The Manitoba IBD Index: evidence for a new and simple indicator of IBD activity. Am J Gastroenterol. 2009;104:1754–1763. 19455122 10.1038/ajg.2009.197

[26] Turner D , Otley AR, Mack D, et al. Development, validation, and evaluation of a pediatric ulcerative colitis activity index: a prospective multicenter study. Gastroenterology. 2007;133:423–432. 17681163 10.1053/j.gastro.2007.05.029

[27] Khanna R , Zou G, D’Haens G, et al. A retrospective analysis: the development of patient reported outcome measures for the assessment of Crohn’s disease activity. Aliment Pharmacol Ther. 2015;41:77–86. 25348809 10.1111/apt.13001

[28] Jairath V , Khanna R, Zou GY, et al. Development of interim patient-reported outcome measures for the assessment of ulcerative colitis disease activity in clinical trials. Aliment Pharmacol Ther. 2015;42:1200–1210. 26388424 10.1111/apt.13408

[29] Herdman M , Gudex C, Lloyd A, et al. Development and preliminary testing of the new five-level version of EQ-5D (EQ-5D-5L). Qual Life Res. 2011;20:1727–1736. 21479777 10.1007/s11136-011-9903-x PMC3220807

[30] Moss AC , Lillis Y, Edwards George JB, et al. Attitudes to mesalamine questionnaire: a novel tool to predict mesalamine nonadherence in patients with IBD. Am J Gastroenterol. 2014;109:1850–1855. 24913040 10.1038/ajg.2014.158

[31] Anderson AJ , Click B, Ramos-Rivers C, et al. Development of an inflammatory bowel disease research registry derived from observational electronic health record data for comprehensive clinical phenotyping. Dig Dis Sci. 2016;61:3236–3245. 27619390 10.1007/s10620-016-4278-z PMC5069178

[32] Narula N , Peerani F, Meserve J, et al. Vedolizumab for ulcerative colitis: treatment outcomes from the VICTORY consortium. Am J Gastroenterol. 2018;113:1345. 29946178 10.1038/s41395-018-0162-0 PMC6445254

[33] Mantzaris G , Bressler B, Kopylov U, et al. DOP55 A real-world comparison of the effectiveness and safety of vedolizumab and anti-TNF therapies in early treatment initiation with first-line biologic therapy in ulcerative colitis: results from EVOLVE. J Crohns Colitis. 2020;14:S092–S094.

[34] Heyman MB , Kirschner BS, Gold BD, et al. Children with early-onset inflammatory bowel disease (IBD): analysis of a pediatric IBD consortium registry. J Pediatr. 2005;146:35–40. 15644819 10.1016/j.jpeds.2004.08.043

[35] Tinsley A , Naymagon S, Trindade AJ, et al. A survey of current practice of venous thromboembolism prophylaxis in hospitalized inflammatory bowel disease patients in the United States. J Clin Gastroenterol. 2013;47:e1–e6. 22476043 10.1097/MCG.0b013e31824c0dea

[36] Shah SC , Naymagon S, Cohen BL, et al. There is significant practice pattern variability in the management of the hospitalized ulcerative colitis patient at a tertiary care and IBD referral center. J Clin Gastroenterol. 2018;52:333–338. 28009685 10.1097/MCG.0000000000000779 PMC6658167

[37] Dalal RS , Vajravelu RK, Lewis JD, et al. Hospitalization outcomes for inflammatory bowel disease in teaching vs nonteaching hospitals. Inflamm Bowel Dis. 2019;25:1974–1982. 31039244 10.1093/ibd/izz089

[38] Bollegala N , Benchimol EI, Griffiths AM, et al. Characterizing the posttransfer period among patients with pediatric onset IBD: the impact of academic versus community adult care on emergent health resource utilization. Inflamm Bowel Dis. 2017;23:1483–1491. 28816756 10.1097/MIB.0000000000001200

[39] Jensen ET , Cook SF, Allen JK, et al. Enrollment factors and bias of disease prevalence estimates in administrative claims data. Ann Epidemiol. 2015;25:519–525.e2. 25890796 10.1016/j.annepidem.2015.03.008 PMC4599703

[40] Prada-Ramallal G , Takkouche B, Figueiras A. Bias in pharmacoepidemiologic studies using secondary health care databases: a scoping review. BMC Med Res Methodol. 2019;19:53. 30871502 10.1186/s12874-019-0695-y PMC6419460

[41] Sands BE , LeLeiko N, Shah SA, et al. OSCCAR: Ocean State Crohn’s and Colitis Area Registry. Med Health R I. 2009;92:82–85, 88. 19385383

[42] Perler BK , Ungaro R, Baird G, et al. Presenting symptoms in inflammatory bowel disease: descriptive analysis of a community-based inception cohort. BMC Gastroenterol. 2019;19:47. 30940072 10.1186/s12876-019-0963-7 PMC6446285

[43] Shapiro JM , Zoega H, Shah SA, et al. Incidence of Crohn’s disease and ulcerative colitis in Rhode Island: report from the Ocean State Crohn’s and Colitis Area Registry. Inflamm Bowel Dis. 2016;22:1456–1461. 26926039 10.1097/MIB.0000000000000745 PMC4868763

[44] Long MD , Kappelman MD, Martin CF, et al. Development of an internet-based cohort of patients with inflammatory bowel diseases (CCFA Partners): methodology and initial results. Inflamm Bowel Dis. 2012;18:2099–2106. 22287300 10.1002/ibd.22895

[45] Neurath MF , Travis SP. Mucosal healing in inflammatory bowel diseases: a systematic review. Gut. 2012;61:1619–1635. 22842618 10.1136/gutjnl-2012-302830

[46] Frøslie KF , Jahnsen J, Moum BA, et al. Mucosal healing in inflammatory bowel disease: results from a Norwegian population-based cohort. Gastroenterology. 2007;133:412–422. 17681162 10.1053/j.gastro.2007.05.051

[47] Lichtenstein GR , Feagan BG, Cohen RD, et al. Serious infections and mortality in association with therapies for Crohn’s disease: TREAT registry. Clin Gastroenterol Hepatol. 2006;4:621–630. 16678077 10.1016/j.cgh.2006.03.002

